# Antibacterial efficacy of endodontic irrigation solutions against *Enterococcus faecalis*

**DOI:** 10.1186/1471-2334-13-S1-P113

**Published:** 2013-12-16

**Authors:** Andreea Cristiana Didilescu, Claudia Melchiori, Luminița Nica, Mihai Săndulescu, Adrian Băncescu, Gabriela Băncescu

**Affiliations:** 1 Faculty of Dental Medicine, Carol Davila University of Medicine and Pharmacy, Bucharest, Romania; 2 Faculty of Dental Medicine, Victor Babeş University of Medicine and Pharmacy, Timişoara, Romania; 3 Faculty of Medicine, Carol Davila University of Medicine and Pharmacy, Bucharest, Romania

## Background

The anatomical complexity of dental root canals represents a major limitation for a successful endodontic treatment, due to the impossibility of complete instrumentation. Therefore, irrigations are required to facilitate removal of microorganisms. The aim of the study was to test the antimicrobial activity of different endodontic irrigants against *Enterococcus faecalis* growth.

## Methods

Forty-one extracted single-rooted teeth were included in the present study. After content removal and autoclaving, they were divided into eight groups among which two were used as positive and negative controls. The remaining six groups were instrumented and irrigated with solutions containing 17% EDTA, sterile saline solution, and different concentrations of NaOCl. In addition, chlorhexidine 2% was also used in three groups. Microbiological evaluation was performed after 30 minutes, and 24 hours, respectively. *E faecalis* strain ATCC 29212 was used for culture tests. Statistical analysis was performed using non-parametric tests.

## Results

NaOCl 6% recorded statistically significant higher antibacterial effect than NaOCl 2.5% (p<0.05). With this regard, no significant differences were recorded between the effects of NaOCl 6% and NaOCl 5.25%. The same outcome was obtained in *E faecalis* growth comparisons between successive dilutions within the same group. Use of chlorhexidine 2% did not influence the results.

## Conclusion

The antimicrobial activity of endodontic irrigants against *E faecalis* was improved by use of higher concentrations of NaOCl (5.25% and 6%).

